# Gas Flow-Dependent Modification of Plasma Chemistry in μAPP Jet-Generated Cold Atmospheric Plasma and Its Impact on Human Skin Fibroblasts

**DOI:** 10.3390/biomedicines11051242

**Published:** 2023-04-22

**Authors:** Dennis Feibel, Judith Golda, Julian Held, Peter Awakowicz, Volker Schulz-von der Gathen, Christoph V. Suschek, Christian Opländer, Florian Jansen

**Affiliations:** 1 Department of Orthopedics Trauma Surgery, Medical Faculty of the Heinrich Heine University, 40225 Düsseldorf, Germany; 2 Plasma Interface Physics, Ruhr University Bochum, 44801 Bochum, Germany; 3 Experimental Physics II, Ruhr University Bochum, 44801 Bochum, Germany; 4 Institute for Electrical Engineering and Plasma Technology, Ruhr University Bochum, 44801 Bochum, Germany; 5 Institute for Research in Operative Medicine (IFOM), Witten/Herdecke University, 51109 Cologne, Germany

**Keywords:** cold atmospheric plasma, hydrogen peroxide, nitrite, nitrate, nitric oxide, inhibition of proliferation

## Abstract

The micro-scaled Atmospheric Pressure Plasma Jet (µAPPJ) is operated with low carrier gas flows (0.25–1.4 slm), preventing excessive dehydration and osmotic effects in the exposed area. A higher yield of reactive oxygen or nitrogen species (ROS or RNS) in the µAAPJ-generated plasmas (CAP) was achieved, due to atmospheric impurities in the working gas. With CAPs generated at different gas flows, we characterized their impact on physical/chemical changes of buffers and on biological parameters of human skin fibroblasts (hsFB). CAP treatments of buffer at 0.25 slm led to increased concentrations of nitrate (~352 µM), hydrogen peroxide (H_2_O_2_; ~124 µM) and nitrite (~161 µM). With 1.40 slm, significantly lower concentrations of nitrate (~10 µM) and nitrite (~44 µM) but a strongly increased H_2_O_2_ concentration (~1265 µM) was achieved. CAP-induced toxicity of hsFB cultures correlated with the accumulated H_2_O_2_ concentrations (20% at 0.25 slm vs. ~49% at 1.40 slm). Adverse biological consequences of CAP exposure could be reversed by exogenously applied catalase. Due to the possibility of being able to influence the plasma chemistry solely by modulating the gas flow, the therapeutic use of the µAPPJ represents an interesting option for clinical use.

## 1. Introduction

It is generally recognized that non-thermal “cold” atmospheric pressure plasma (CAP) has great potential for numerous technological as well as medical-therapeutic applications [1,2]. A common feature of such plasma is the reduced spatial dimension (from a few microns up to a few millimeters) of the confining structures, e.g., electrodes, stabilizing the discharge and preventing the transition to a “thermal” arc discharge. These types of generated discharges are often summarized as “microplasmas” and contain high concentrations of radicals at low gas temperatures [3,4,5]. Therefore, microplasma-producing jets as the source are suitable for many applications, in particular, the modifications of sensitive surfaces and plasma medicinal applications, including sterilization, the treatment of cancer and impaired wound healing [6,7,8]. A microplasma-producing jet often uses helium as the carrier gas, and numerous investigations of RF plasma jets and their potential clinical use have been conducted [9,10,11,12,13,14,15,16,17,18].

Many research groups around the world use technically diverse and mostly self-designed and developed atmospheric pressure plasma jets to establish biomedical applications [19,20,21,22]. This complicates comparisons between studies conducted with the different plasma sources and greatly delays exploration and insight into the fundamental understanding of CAPs and their interaction with biological structures [23]. This in turn slows down the national and international approval procedures for this technology for medical applications [24]. As part of the European Cooperation in Science and Technology (COST)-Action MP1011, the microscale atmospheric pressure plasma jet (μAPPJ) developed by Schulz-von der Gathen and colleagues was selected as the basis for the development of a reference source to solve these problems [25,26], the COST reference microplasma jet [27,28,29]. For the µAPPJ, small admixtures of oxygen and additional nitrogen contributions from surrounding air result in the generation of reactive oxygen nitrogen species (RONS), such as ozone (O_3_), hyperoxide (O_2_^−^), hydroxyl radicals (-OH) as well as nitric oxide (NO) and nitrogen dioxide (NO_2_) [2,15,26,30,31]. In particular, NO regulates many processes in human skin physiology [32,33,34] and many pathological skin conditions, including psoriasis, impaired wound healing microcirculation, and skin tumor formation, are also associated with an imbalance in NO-biosynthesis [35,36,37,38]. Thus, the µAPPJ device could be a tool in treating different skin and wound conditions with cold atmospheric-pressure plasma (CAP) through the delivery of NO or bioactive NO compounds and the induction of NO-dependent pathways, as already shown for other plasma sources by several studies [39,40,41]. It has been shown in numerous studies that CAP can induce severe damage up to cell death in a large number of mammalian cells, including skin and blood cells, which is a disadvantage per se when using plasma to treat chronic and acute wounds or inflammatory wounds, which can represent skin diseases [42,43,44,45,46,47,48].

In cancer, the induction of apoptotic (programmed) or necrotic cell death by CAP could be an excellent and attractive therapeutic tool [49,50,51,52,53,54,55,56,57]. Many different plasma devices were used in these and other studies, but the observed CAP-induced cell toxicity correlated positively with plasma treatment time or plasma dose independent of the CAP device used [43,44,50,52,56,58,59,60,61].

Lower doses or shorter treatment time showed lesser toxicity as well as many interesting biological effects, such as enhanced proliferation of porcine endothelial cells by short CAP exposure (30 s) using a dielectric barrier discharge (DBD) device, correlated with a significant release of FGF-2 [43]. By using a KINpen plasma jet as the CAP source, a treatment of HaCaT (30 s) reduced the cell number and down-regulated E-cadherin and EGF receptor expression, whereas no effects were observed after shorter treatment time (10 s) or after changing the media after treatment [59]. Using a microwave plasma torch as the CAP source, Arnd et al., 2013, described a long-term inhibition of the proliferation of melanoma cells after 1 min and toxic effects after 2 min of treatment [50]. It is believed that the oxidative effects of reactive oxygen/nitrogen species (ROS/RNS) are responsible for the observed plasma-induced effects, since the presence of antioxidants can positively influence the toxic plasma effects [55,62]. However, the survival and functionality of cells depends, among other things, on a humid milieu, with constant physiological pH value, temperature and osmolarity, and the sufficient supply of nutrients and oxygen. In this context, plasma jets can lead to dehydration effects in vitro and in vivo due to the relatively high gas flows of up to 6 slm, which, for example, can also severely limit the treatment times with such devices. In comparison, the µAPPJ operates with gas flows between 0.25 and 1.4 slm, which allows longer treatment times and limits potential desiccation effects.

In addition, plasma treatment can acidify cell culture media and other exposed aqueous solutions, might increase nitrite and nitrate concentrations [52,57,58,60,63], and can lead to the formation of hydrogen peroxide (H_2_O_2_) [58,64], all of which are known to mediate cellular responses and may be responsible for many of the observed CAP-induced effects.

Human skin fibroblasts (hsFB) play a central role in the regulation and maintenance of wound healing. In the process of wound healing, hsFB-relevant cytokines proliferate and synthesize and form a provisional extracellular matrix (ECM) by generating collagen and fibronectin [65,66]. Regarding the small volume of liquids in wounds, CAP sources may induce many biological responses via chemical and physical modifications of the liquid microenvironment of cells, in addition to the generation of reactive oxygen/nitrogen species. Therefore, the current study aims to investigate the effects of µAPPJ treatment on the viability and proliferation of human dermal fibroblasts, elucidating the role of possible CAP-induced chemical and physical changes in the treatment medium, such as an accumulation of NO-related compounds and H_2_O_2_, as well as acidification and an increase in osmolarity.

## 2. Materials and Methods

### 2.1. Plasma/CAP Source

The μAPPJ is a capacitively coupled microplasma jet consisting of two stainless steel electrodes (length 30 mm and width 1 mm) with a gap of 1 mm. The plasma is ignited in this gap, filling a volume of 1 × 1 × 30 mm^3^. The complete device consists of the electrode stack with 1.5 mm thick quartz panes enclosing the plasma volume (identical to the COST reference electrode head [28]), the jet holder containing the electric connection, the gas connector and the gas tubing. One electrode is connected to a power supply (13.56 MHz, <1 W) and the other one is grounded. The μAPPJ can be operated for helium gas flows from 0.25 slm up to several slm. Furthermore, small admixtures of molecular oxygen (>0.6%) are possible [15]. The helium flow is controlled by a mass flow controller (FC280S, Millipore-Tylan, Bedford, MA, USA). For all measurements, the μAPPJ was operated at 13.56 MHz and at a helium gas flow of either 0.25 slm or 1.40 slm (helium purity 99.999%, Linde, Munich, Germany).

In anticipation of atmospheric impurities in the process gas, e.g., in the form of traces of water, oxygen and/or nitrogen from the ambient air, which should lead to a higher yield of reactive oxygen species and nitrogen oxide species of the produced CAPs, we exchanged the metal gas lines of the processing gas supply with silicone gas lines. With this procedure, we were fully aware that such a procedure could not generate a previously defined degree of the aforementioned contamination of the processing gas. However, a detailed control of the admixtures, especially with very small gas flows, was unfeasible. The method we used was intended to demonstrate the principle involved. N_2_, O_2_ and H_2_O would always be involved in small amounts from the ambient air in any treatment.

**Figure 1:**
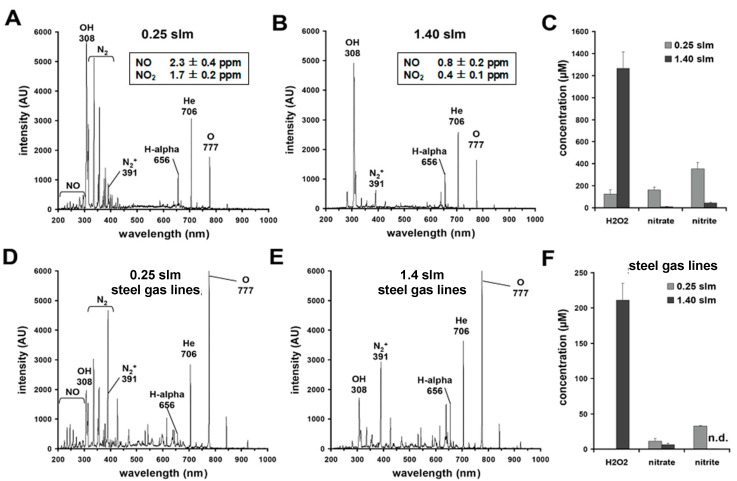
Gas line material affects plasma composition and the accumulation rate of H_2_O_2_, nitrate and nitrite. OES-spectra of µAPPJ plasma generated at different gas flows (0.25 slm (**A**); 1.4 slm (**B**)) using silicon gas lines for gas delivery. (**C**) The plasma-induced generation of hydrogen peroxide (H_2_O_2_), nitrate and nitrite found in PBS (500 µL) after a single plasma treatment (10 min) under the same conditions. By replacing the silicon gas lines by steel gas lines, different OES spectra were obtained (**D**,**E**). The H_2_O_2_, nitrate and nitrite concentrations found in PBS (500 µL) after CAP treatment (10 min) using steel gas lines are shown in (**F**). Bars shown in (**C**,**F**) represent the mean ± SD of three individual experiments.

Optical emission spectroscopy (OES, HR 4000, Ocean Optics, Duvien, The Netherlands) using an optical fiber was applied to characterize the plasma under experimental conditions at about 1 mm from the tip of the jet electrode (Figure 1).

### 2.2. Determination of Evaporation and Temperature

As preliminary work, the evaporation rate was estimated by measuring the liquid volumes (250, 500, 750, 1000 µL) in cell culture plates (24-well) before and after μAPPJ treatments or gas control (flow rates 0.25 slm or 1.4 slm) by a pipette (Eppendorf, Wesseling, Germany). In the preliminary experiments, the temperature of treated buffer was measured by using a digital thermometer (GMH3230, Greisinger Electronic, Regenstauf, Germany) after different treatment intervals (0, 5, 10 min).

### 2.3. Determination of pH Values

The pH values were determined before and after the plasma treatment using a calibrated pH meter (Calimatic 766, Knick, Berlin, Germany) and a corresponding pH electrode from Mettler-Toledo (Giessen, Germany).

### 2.4. Measurement of Dissolved Oxygen

The oxygen saturation/concentration of the treated and untreated buffer (500 µL/24-well plate, 0–10 min) at different gas flows (0.25 and 1.4 slm) was measured by using a multiparameter pH-Meter (HI2020-edge, Hanna Instruments, Carrollton, TX, USA) and a digital dissolved oxygen/temperature electrode (HI764080, Hanna Instruments, Carrollton, TX, USA).

### 2.5. Detection of Nitrite and Nitrate

The nitrite concentrations were quantified by an iodine/iodide-based assay using the NO analyzer CLD 88 from Ecophysics (Munich, Germany), and the determination of the total concentration of nitrite and nitrate was carried out using the vanadium (III) chloride method as previously described [67,68,69].

### 2.6. Measurement of H_2_O_2_

The concentration of H_2_O_2_ in CAP-treated PBS was determined by the titanium oxide oxalate method as previously described [70].

### 2.7. Measurement of Nitric Oxide and Nitrogen Dioxide

The determination of nitrogen monoxide (NO) and nitrogen oxides (NO_2_) in the gas phase at the outlet of the μAPPJ was measured using the NO/NO_x_ analyzer CLD 822r (Ecophysics, Munich, Germany).

### 2.8. Cell Culture

Primary cultures of hsFB were isolated from abdominoplasty skin specimens obtained with donor consent and approval of the Ethics Commission of Düsseldorf University (Study No. 3634) from 7 female and 1 male patients (39–75 years old, mean 52.4 ± 15.3 years).

The hsFB cultures were isolated, cultivated and cryopreserved as previously described [71]. For the experiments, cryopreserved primary hsFB cultures were thawed and cultivated in T75 cell culture flasks (Cellstar, Greiner Bio-One, Frickenhausen, Germany) at 5% CO_2_ and 37 °C. For seeding, the cells were detached from the surface by adding 0.05% trypsin/0.02% EDTA/0.9%, and the remaining trypsin activity was neutralized by adding 1 mL fetal calf serum (FCS). hsFB cultures were grown in Dulbecco’s modified Eagle’s medium (DMEM, Gibco-Invitrogen, Karlsruhe, Germany), plus 10% FCS (SeraPlus, Pan-Biotech, Aidenbach, Germany), 100 U/mL penicillin and 100 µg/mL streptomycin (PAA, Pasching, Austria) cultivated. Experiments were carried out in 24-well cell culture plates with a cell density of 2.5 × 10^4^ per well. All measurements were performed with hsFB from passages 4–6.

### 2.9. Plasma Treatments of Fibroblasts

**Figure 2:**
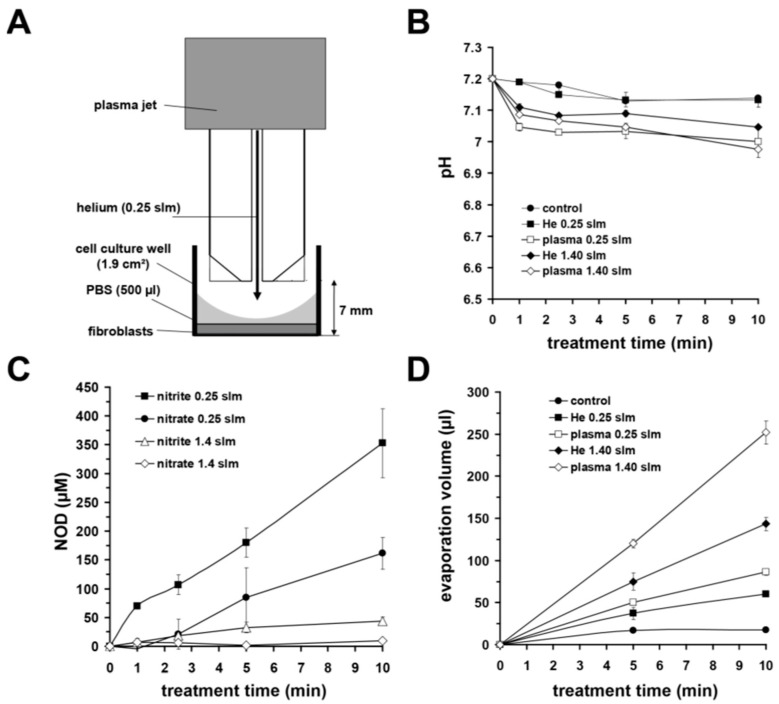
Experimental set-up and plasma-induced modifications of buffer. (**A**) Experimental set-up for µAPPJ treatments. (**B**) Evaluated plasma-induced pH changes. (**C**) Accumulation of nitrite and nitrate by plasma treatment obtained with different gas flows. (**D**) Loss of buffer volume after plasma/gas application using different gas flows. Values represent the means ± SD of five individual experiments.

Prior to plasma treatment in PBS (500–1000 µL), the hsFB were carefully washed (PBS, 500 µL), and the culture plate was placed under the μAPPJ. The distance between the electrode end and the culture plate well’s bottom in all experiments was kept at 7 mm (Figure 2A) for direct treatments. For indirect treatments, the buffer or media was separately treated from the hsFB and immediately transferred to the hsFB-containing cell culture plate. The directly and indirectly treated hsFB were incubated as indicated (0–5 min) before buffer was removed and fresh media was added. In addition, the hsFB were incubated with a buffer containing bovine catalase (1000 U/mL, Sigma Aldrich, St. Louis, MO, USA).

### 2.10. Toxicity, Viability and Proliferation

We used three vital dyes to detect and quantify cytotoxic events as described previously [72,73]. Vital cells were detected using fluorescein diacetate (FDA), apoptotic cells were visualized and quantified using Hoechst 33342 dye and we used propidium iodide to detect and quantify necrotic events. The fluorescent dyes were each used in a concentration of 0.5 μg/mL. The experiments were evaluated using a fluorescence microscope (Zeiss, Wetzlar, Germany). In addition, we characterized the vitality and cell number in the respective differently treated cell cultures using a resazurin-based assay (CellTiter-Blue, Promega, Madison, WI, USA) and using a fluorescence spectrometer (VICTOR II Plate Reader, PerkinElmer, Waltham, MA, USA) at an excitation wavelength of 540 nm and an emission wavelength of 590 nm, as described previously [72,73].

### 2.11. Statistical Analysis

Significant differences were evaluated by using either paired two-tailed Student’s *t*-test or ANOVA followed by appropriate post hoc multiple comparison tests (Tukey method). A value of *p* < 0.05 was considered as significant.

## 3. Results

### 3.1. Plasma Characterization

The comparison of OES spectra taken with different gas flows and supply lines is shown in Figure 1.

In Figure 1A,B, we show the OES spectra of µAPPJ plasma at gas flows of 0.25 slm and 1.4 slm using silicon gas lines for gas supply, while Figure 1D,E shows the spectra after replacing silicon lines with steel lines. Irrespective of the chosen gas flow values, after change of the gas lines, apart from the expected signals of helium, other dominant signals appeared, which were identified as impurities. At 0.25 slm (Figure 1A), in addition to signals from atomic hydrogen, neutral/ionic molecular nitrogen and nitric oxide, a marked peak at 308 nm can be observed, indicating the occurrence of OH in the plasma. This is most probably the result of enhanced humidity in the system’s gas line of silicon due to stored moisture as well as greater diffusion from the immediate environment. At a gas flow of 1.40 slm (Figure 1B), the signals of NO and neutral nitrogen molecules are much lower.

Additionally, Figure 1C,F shows the concentration of H_2_O_2_, nitrate and nitrite, which were obtained after a plasma treatment (10 min) of PBS (500 µL) under the respective gas flow conditions.

Using the silicone gas lines, the 10 min treatment of the aqueous solutions, regardless of the gas flow used, resulted in a significant and many times higher accumulation of H_2_O_2_, nitrite and nitrate (Figure 1C) than when using the µAPPJ with the steel gas lines (Figure 1F). Furthermore, as we show in the inserted tables in Figure 1A, B, we were able to detect considerable amounts of nitric oxide (NO) and nitrogen dioxide (NO_2_) in the μ-APPJ plasmas using the CLD technique. The measured NO and NO_2_ concentrations at a lower gas flow (0.25 slm) were 2.3 ± 0.4 ppm and 1.7 ± 0.2 ppm, respectively, whereas at a gas flow of 1.40 slm, NO achieved only 0.8 ± 0 0.2 ppm and NO_2_ 0.4 ± 0.1 ppm. Based on these results, all of the following experiments were conducted using silicone gas supply lines.

### 3.2. Impact of CAP on Physicochemical Modifications of Aqueous Solutions

Using an experimental setup, as shown in Figure 2A, we found that a treatment of buffer with μAPPJ plasma led to a slight, albeit significant, reduction in pH (Figure 2B) and to a partly strong evaporation, particularly at the higher gas flow (Figure 2D). We observed that after plasma treatment (10 min), the volume loss of the treated buffer at a 1.4 slm gas flow was almost 50%. In contrast, using 0.25 slm, the loss of volume was about 17% of the initial volume. Without plasma ignition, the helium gas flow resulted in volume losses of ~29% (at 1.40 slm) and ~12% (at 0.25 slm) without plasma (Figure 2D).

As shown in Figure 2C, the chosen gas flow also had a modulative effect on the nitrite and nitrate concentrations in µAPPJ-plasma exposed buffers. At the gas flow of 0.25 slm, we were able to detect nitrite (161.4 ± 27.7 µM) and nitrate (352.7 ± 59.9 µM) after plasma treatment (10 min), whereas the nitrite concentration in the treated buffer was 9.7 ± 2.4 µM, and the nitrate concentration was 44.1 ± 6.6 µM at 1.40 slm (Figure 2C).

**Figure 3:**
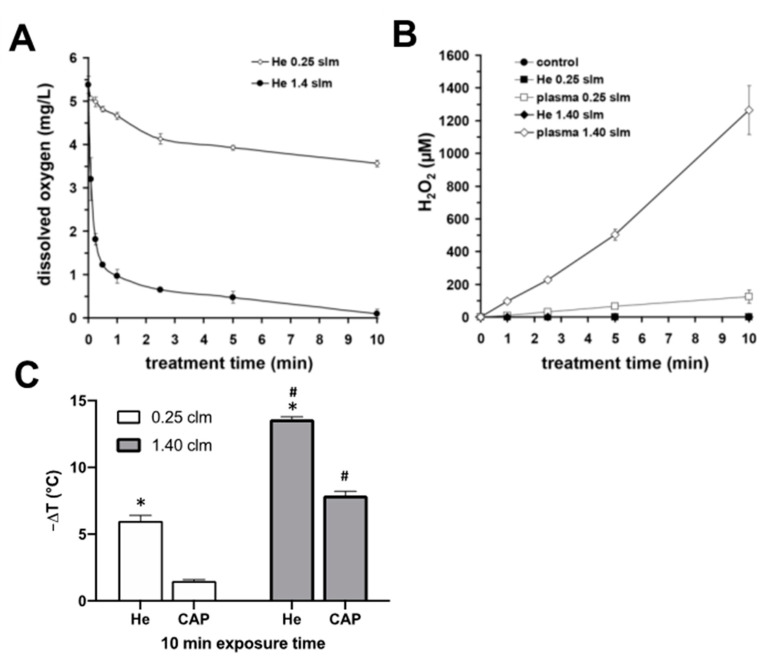
Plasma-induced modifications of buffer. (**A**) The gas flow-induced changes of dissolved oxygen in the buffer. (**B**) Accumulation of hydrogen peroxide by plasma treatment using different gas flows. Values represent the means ± SD of five individual experiments. (**C**) Decrease in temperature (−∆T) of gas (He) or plasma (CAP) exposed aqueous solutions using gas flows of 0.25 slm or 1.40 slm. (**A**–**C**) Values represent the mean ± SD of four individual experiments. * *p* < 0.05 as compared to CAP-treatment; **^#^** *p* < 0.05 as compared to respective values obtained with 0.25 slm.

We also evaluated the influence of the gas flow rate on the oxygen content of the He-exposed (10 min) solution and found, as expected, a significantly greater oxygen depletion of the treated solution with the treatment with 1.40 slm compared to the treatment with 0.25 slm (Figure 3A).

Characterizing the ability of μAPPJ to generate H_2_O_2_ in exposed buffer solutions, we observed a time-dependent linear H_2_O_2_ production rate in the treated solutions (Figure 3B). With a gas flow of 1.4 slm, we observed an H_2_O_2_ concentration of 1265 ± 148 µM after 10 min of exposure and at 0.25 slm, we quantified an approximately 10-fold lower H_2_O_2_ concentration of 124 ± 40 µM.

The gas flow we used also had a significant effects on the temperature of the CAP-treated buffer. After treatment (10 min, 1.40 slm) with helium (without plasma ignition), the temperature of the buffer dropped by 13.6 ± 0.2 °C and by 7.9 ± 0.3 °C with the helium plasma ignited. The cooling effects mentioned were −6.0 ± 0.4 °C in the case of helium gas exposure alone (without plasma ignition) and −1.5 ± 0.1 °C after CAP exposure (10 min) using 0.25 slm (Figure 3C).

### 3.3. Impact of Helium Stream Exposure on Viability of Primary Human Skin Fibroblasts

**Figure 4:**
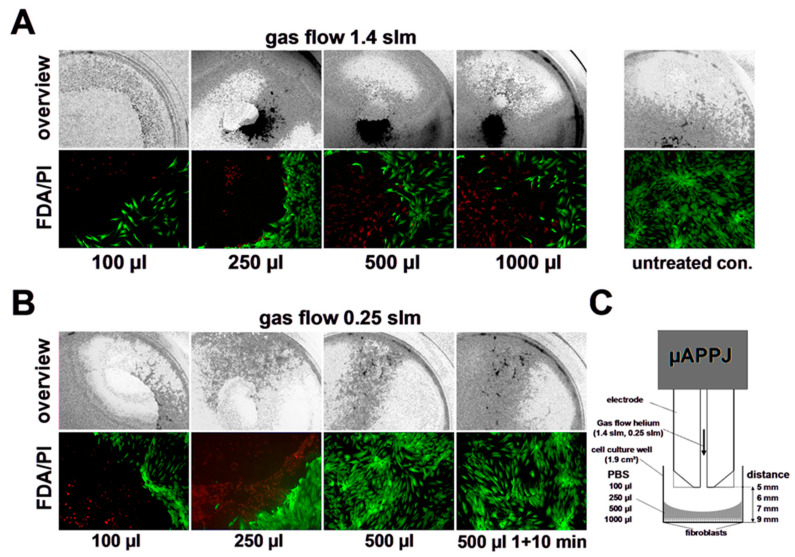
Helium treatment induced toxicity. Representative microscopy images of human skin fibroblasts cultures and their respective fluorescence images (fluorescein diacetate, FDA/propidium iodide, PI) directly after helium treatment (10 min) at different gas flows (1.40 slm (**A**); 0.25 slm (**B**)) applied by the µAPPJ without plasma ignition (**C**) and treatment volumes (as indicated) of buffered phosphate saline (PBS).

Based on some of the phenomena mentioned above, one can assume that just a physical modification of a solution by the gas flow used, e.g., a lowering of the oxygen content, the reduction of the medium volume and the resulting increased osmotic stress, has a negative effect on the vitality of the exposed cell cultures. To verify this assumption, we overlaid human fibroblast cultures in cell culture dishes with medium (100–1000 µL) and exposed them to a 1.40 or 0.25 slm flow of helium for 10 min via the µAPPJ, but without plasma ignition. As shown in Figure 4A, treatment with 1.40 slm led to a significant increase in toxicity, which correlated with decreasing medium volumes of the respective cell culture. This observed toxic effect of the helium flow was significantly reduced when using the lower 0.25 slm gas flow (Figure 4B).

### 3.4. Impact of CAP on Viability of Primary Human Skin Fibroblasts

In addition to direct CAP exposure, as shown in Section 3.2 and Section 3.3, to avoid the subsequent dehydration effects caused by the carrier gas flow, the fibroblast cultures were “indirectly” treated with the plasma. In this case, the buffer was treated with CAP separately and immediately added to the fibroblast cultures for further incubation (0, 1, 3, 5 min). Alternatively, in order to estimate the possible osmotic effects, the CAP-induced loss of buffer volume as a result of the treatment was compensated by adding the respective evaporated amount of water.

**Figure 5:**
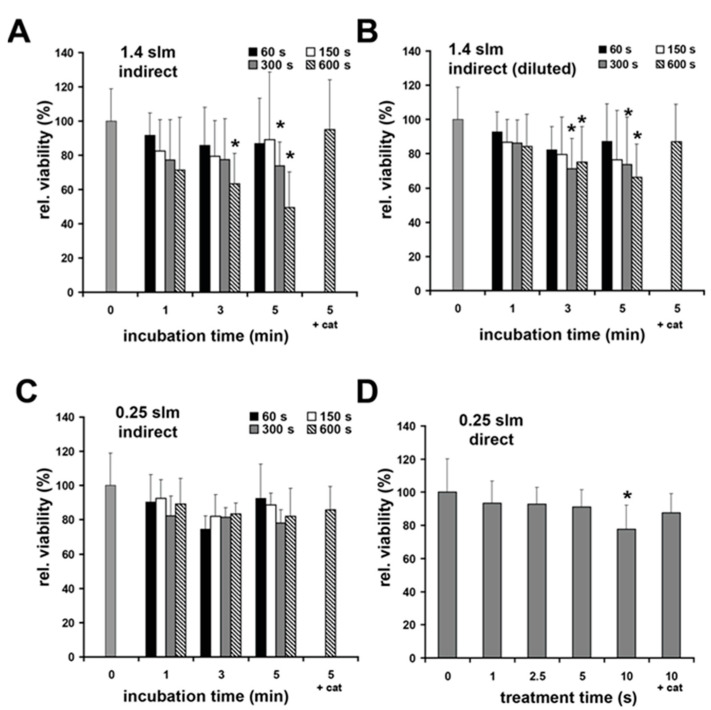
Plasma effects on cell viability. (**A**) Cell viability of human skin fibroblasts measured by a resazurin-based assay 24 h after indirect plasma treatment (gas flow 1.4 slm) followed by short incubation as indicated, (**B**) 24 h after indirect plasma treatment (gas flow 1.4 slm) followed by short incubation as indicated with prior compensation of plasma-induced loss of buffer, (**C**) 24 h after indirect plasma treatment (gas flow 0.25 slm) followed by short incubation as indicated, and (**D**) 24 h after direct plasma treatment (gas flow 0.25 slm). Catalase (1000 U; +cat) was added for a treatment time of 600 s followed by 5 min incubation. Catalase and gas treatments alone did not show significant effects on cell viability (not shown). Values represent the means ± SD of 7–8 independent experiments. * *p* < 0.05 as compared to the control values (0 min incubation time).

In Figure 5A, we show the results of indirect treatment of the fibroblast cultures with the µAPPJ-generated CAP at 1.40 slm without compensating for the volume loss. Here, it can be seen that the toxic effect of the treatment correlates with the length of the treatment time of the cell culture medium and the exposure time of the cells to this medium.

We were able to determine a significant decrease in cell vitality after treatment of cell cultures with CAP-exposed (10 min at 1.40 slm) buffers to 49 ± 29% of the vitality of the original culture. However, the toxic effect of indirect treatment of cell cultures was significantly weaker when the volume loss of CAP-treated buffer was replenished to the initial volume by the addition of water before distribution to cell cultures was performed (Figure 5B). It should be kept in mind that the final H_2_O_2_ concentration (600 s treatment, 5 min incubation) documented in Figure 5A was again reduced approximately two-fold after volume compensation (Figure 5B). The addition of catalase (+cat) resulted in a significant mitigation of the toxic effect of indirect CAP treatment of cell cultures, further underscoring the dominant role of H_2_O_2_ in the experiment described above.

After indirect plasma treatments using 0.25 slm gas flow, no significant effects on cell viability were observed, regardless of the presence of catalase in the CAP-exposed buffer (Figure 5C). After direct plasma-treatment (0.25 slm), we observed a slight, albeit significant, reduction in cell viability (down to 77 ± 15%) only after a 10 min treatment, which was significantly reduced by the addition of catalase (Figure 5D). Treatment with helium as a control did not show significant differences to the untreated control (96 ± 14, 0.25 slm; 95 ± 13%, 1.4 slm).

### 3.5. Impact of CAP on Proliferation Capacity of Primary Human Skin Fibroblasts

**Figure 6:**
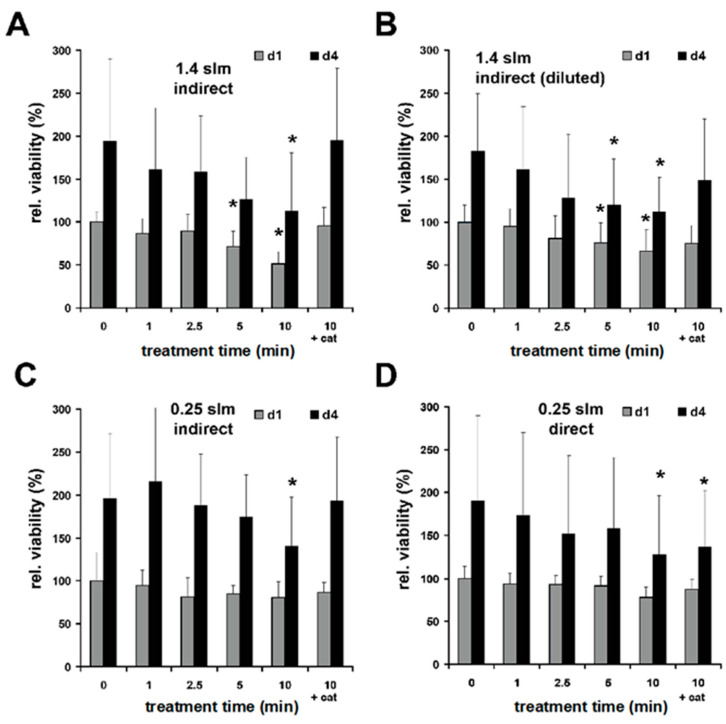
Plasma effects on cell proliferation. The cell numbers of human skin fibroblasts normalized to the untreated control were measured on d1 and d4 after plasma treatment by a resazurin-based assay. (**A**) Indirect plasma treatment (gas flow 1.4 slm) followed by 5 min incubation. (**B**) Indirect plasma treatment (gas flow 1.4 slm) followed by 5 min incubation with prior compensation for buffer’s plasma-induced loss. (**C**) Indirect plasma treatment (gas flow 0.25 slm) followed by 5 min incubation. (**D**) Direct plasma treatment (gas flow 0.25 slm). Catalase (1000 U; cat) was added for 600 s treatment followed by 5 min incubation. Catalase and gas treatments alone did not show significant effects on cell numbers (not shown). Values represent the means ± SD of 7–8 independent experiments; * *p* < 0.05 as compared to the control values (0 min incubation time).

The results documented in Figure 6A,B also show that the proliferation of cell cultures treated indirectly with CAP (1.4 slm) followed by a 5 min incubation was significantly inhibited when volume compensation was omitted. Here, too, this effect could be significantly reduced by the addition of catalase (+cat), which in turn points to a CAP-induced H_2_O_2_ accumulation as the cause of the reduction in the proliferation rate.

Direct and indirect treatment (10 min, 0.25 slm) reduced the cell numbers on day one and day four as compared to the controls (Figure 6C,D). By the addition of catalase, the observed inhibition of proliferation could be reserved only for indirect treatment (Figure 6C).

## 4. Discussion

For more than two decades, modern plasma technology has allowed the generation of “cold” atmospheric plasmas (CAP), even under atmospheric pressure conditions and low process temperatures, which enables interactions with biological tissues. Based on good tolerability, there are a variety of plasma-based therapy options for the treatment of different diseases in humans and animals [74,75]. Depending on the technology used and the atmospheric environment, CAPs contain reactive nitrogen species (RNS), such as nitric oxide (NO), nitrogen dioxide (NO_2_), reactive oxygen species (ROS and ozone (O_3_)), superoxide radicals (.O_2_^−^), hydroxyl radicals (.OH) and many other radical products in different concentrations and compositions [76,77]. The components contained in CAPs, individually or in combination, can affect the biological functions of tissues and cells [41]. Nitric oxide and some of its derivatives have an important physiological function in the regulation of inflammatory response, vasodilation, angiogenesis, thrombogenesis, immune response, cell proliferation/differentiation, antibacterial defense, collagen metabolism, apoptosis and necrosis [78]. The pivotal importance of NO in the regulation of tissue homeostasis becomes particularly clear in situations characterized by insufficient NO production or NO availability. A relative and absolute NO deficiency correlates with the corresponding chronic, bacterially infected and poorly healing wounds seen in the clinic [79].

In this respect, it is not surprising that the positive effect of NO-based therapies with exogenously applied NO gas, NO donors or NO-containing plasmas in the therapy of chronic wounds have been shown in various studies [80,81,82]. In addition, the bacterial infection of wounds is a driving factor in delayed wound healing, and all measures that lead to a reduction of the bacterial burden may improve the wound healing status. Therefore, plasma compositions, in particular those characterized by dominant H_2_O_2_ production, were shown to be very effective in combating the bacterial load on the wound and support wound healing in a particularly positive way [83]. It is therefore expedient to generate plasmas with different properties adapted to the desired therapy goals by modulating the plasma chemistry. A particular bacteriotoxic effect could be aimed at using H_2_O_2_-enriched or H_2_O_2_-generating plasmas, whereas the modulation of NO-dependent physiological parameters could be achieved most likely with strongly NOD-generating plasmas.

With a DBD device, we very recently observed that by increasing the power dissipation in the discharge, it was possible to shift from a plasma chemistry characterized by oxygen radicals towards a nitrogen oxide–dominated chemistry [84]. Under the conditions given here, i.e., in the presence of the atmospheric impurities mentioned, such a shift in plasma chemistry could be achieved with the µAPP-Jet simply by selecting the flow rate of the operating gas (Figure 1C,E). With the “regular” use of the plasma source, i.e., the operating gas supplied via stainless steel lines [85], oxygen radical-dominated plasmas could be generated at higher flow velocities (1.40 slm), and at lower flow velocities (0.25 slm) plasmas predominantly dominated by nitrogen oxides are generated. Establishing a reference jet based on μAPPJ technology (COST jet; see introduction), Schulz-von der Gathen and colleagues found that the material of the gas lines can strongly influence the reproducibility and the purity of the generated plasmas. Here, even the slightest contamination of the process gas with air, oxygen and/or H_2_O could lead to a significant change in the composition of the resulting plasmas [28,29].

With this knowledge, in our experiments we used silicone tubes instead of metal tubes. The intention was to provoke contamination and thereby achieve a higher yield of reactive species in the plasma. Our technical equipment did not allow us to actively and specifically control the degree of contamination of the process gas, but the optical emission spectroscopy (OES) analysis revealed a defined time-constant level of specific impurities with the use of silicone tubes. In the OES diagram, in addition to NO, neutral/ionic molecular nitrogen and atomic hydrogen also the presence of OH radicals in the form of a clear peak at 308 nm could be seen, most likely representing the result of increased humidity in the tube atmosphere. However, this supposedly low level of contamination had serious consequences for the composition and chemical properties of the plasma. Using the silicone tubes, plasma exposure of aqueous solutions led to the accumulation of six times the H_2_O_2_ concentration and an almost 16-fold higher NOD concentration compared to the µAPPJ plasmas driven with metal tubes. Additionally, regardless of the significantly higher plasma concentrations of the reactive species when using the silicone gas lines, we observed up to 20-fold higher production rates of NO derivatives in the plasma-exposed solutions when using the lower gas flow of 0.25 slm than with 1.40 slm. In contrast, up to 14 times higher H_2_O_2_ concentrations were obtained in the CAP-treated solutions using 1.40 slm than in those using 0.25 slm. A possible and the most reasonable explanation for this phenomenon is related to the very short lifetime of the generated oxygen atoms due to their large reaction potential. It can be assumed that with a low gas flow, the generated reactive oxygen species may not even reach the exposed object and react quickly to the corresponding NOX if N is present. At high flux levels, one would accordingly expect a greater influence of the ROS chemistry.

The simple option of the µAPPJ, which involves modulating the flow rate of the processing gas to decisively change the plasma chemistry in the direction of a ROS- or RNS-directed therapy option, has a major advantage over other plasma-device technologies, such as DBD technology. For example, the preference for plasma-induced nitrite accumulation and acidification is an essential aspect supporting wound healing and other NO-dependent physiological processes [86] but also an important reinforcing mechanism in the antibacterial effect of NOD-containing or NOD-generating plasma [63]. On the other hand, an effective therapeutic cytotoxic effect against cells and bacteria could be achieved through a preferred plasma-induced formation of the strong oxidizing agent H_2_O_2_ [58,64]. Irrespective of the injurious H_2_O_2_-induced effects on different cell types described in the literature [87,88],we were only able to observe very low cytotoxic effects, even with high H_2_O_2_ concentrations, with the human skin fibroblasts investigated here. These cell-damaging effects of the µAPPJ-induced H_2_O_2_-generating plasma could also be completely avoided by the exogenous addition of catalase. In addition to the change in plasma chemistry described above by modulating the gas flow, it is technically possible to change the plasma chemistry in a targeted and defined manner by adding the appropriate gas species in a controlled manner.

Another relevant aspect in the therapeutic use of plasma jet technology is the flow rate of the processing gas required for optimal plasma generation. Every cell requires a certain moist environment with a controlled physiological pH and physiological osmolar pressure, a suitable temperature and an adequate supply of oxygen and nutrients for its survival and the exercise of physiological functions. In particular, plasma jets with very high gas flow rates of up to 6 slm could increase the osmolarity and greatly reduce the temperature due to dehydration effects and reduce the oxygen supply to the cells due to the relatively high concentration of the operating gases. Loss of volume through evaporation and simultaneous accumulation of the NOD and/or H_2_O_2_ concentration can lead to hyperosmolarity and all the associated negative effects [89,90], including cell death. Since the µAPPJ can also be operated with incomparably low flow rates compared to other plasma jet devices using different plasma generation technologies, the described negative influences of a high gas flow could be completely neglected.

In summary, the µAPP-Jet can be operated with a comparably wide variation in the flow rate of the processing gas. In particular, a possible operation of the device at low gas flow rates takes into account the problem of dehydration of the exposed areas or samples, as can be observed when using plasma devices operated with very high gas flow rates. With the µAPP-Jet, a low operating gas flow rate correlates with a dominant NO chemistry of the generated plasma, whereas a dominant oxygen radical chemistry can be controlled at high flow rates of the processing gas. Through the targeted and defined introduction of traces of air gases or water, the concentration of ROS and RNS in the generated plasma can be increased many times over, with the above-mentioned effects of the gas flow remaining intact. The properties mentioned make the µAPPJ technology very attractive for novel therapy options in the treatment, e.g., bacteria-infested, poorly healing wounds. By choosing the flow rate of the processing gas, NOD-based support of the physiological processes of wound healing can be addressed and ROS-based therapy goals of bacterial disinfection of a wound can be promoted.

## Data Availability

The data that support the findings of this study are available from the corresponding author upon reasonable request.
